# Sensing Landscape History with an Interactive Location Based Service

**DOI:** 10.3390/s90907217

**Published:** 2009-09-09

**Authors:** Ron van Lammeren, Martin Goossen, Paul Roncken

**Affiliations:** 1 Wageningen University/Geo Information Science and Remote Sensing, P.O. Box 47, 6700AA Wageningen, The Netherlands; 2 Alterra / Centre Landscape, P.O. Box 47, 6700AA Wageningen, The Netherlands; E-Mail: Martin.Goossen@wur.nl; 3 Wageningen University/Landscape Architecture, P.O. Box 47, 6700AA Wageningen, The Netherlands; E-Mail: Paul.Roncken@wur.nl

**Keywords:** human sensor, informal interactive Location Based Services, photo classification, sensing cultural-historic objects, spatial density, STEAD approach

## Abstract

This paper introduces the STEAD approach for interpreting data acquired by a “human sensor”, who uses an informal interactive location-based service (iLBS) to sense cultural-historic facts and anecdotes of, and in the landscape. This user-generated data is collected outdoors and *in situ*. The approach consists of four related facets (who, what, where, when). Three of the four facets are discussed and illustrated by user generated data collected during a Dutch survey in 2008. These data represent the personal cultural-historic knowledge and anecdotes of 150 people using a customized iLBS for experiencing the cultural history of a landscape. The “who” facet shows three dominant mentality groups (cosmopolitans, modern materialists and post modern hedonists) that generated user content. The “what” facet focuses on three subject types of pictures and four picture framing classes. Pictures of the place type showed to be dominant and foreground framing class was slightly favourite. The “where” facet is explored via density, distribution, and distance of the pictures made. The illustrations of the facets indirectly show the role of the “human sensor” with respect to the domain of interest. The STEAD approach needs further development of the when-facet and of the relations between the four facets. Finally the results of the approach may support data archives of iLBS applications.

## 1. Introduction

Location-Based Services (LBS) are a booming topic in business [1] and science [2]. They are defined as “geographically-oriented data and information services to users across mobile telecommunications networks” [3]. Contemporary devices combined with software developments based on Web 2.0 extend LBS into the direction of *interactive Location-Based Service* (*iLBS*). With *iLBS*, the user of a mobile smart-phone is no longer simply the consumer of location and of context-dependent information via push and pull interfaces, but also becomes a participant in collecting data and reviewing information. *Interactive LBS* constitutes the next stage – locative media [4] – in the use of mobile phone technology. The current generation mobile phones provide many functions to acquire location-based data (via GPS) by text files, digital pictures, sound files and video streams. These data can be gained at will and can be stored and shared via wireless internet. Therefore, we define *iLBS* as “geographically-oriented data and information services to support user communities across mobile telecommunications networks”.

One could envisage that people equipped with such mobile devices and linked to an *iLBS* could serve as part of a sensor network [5]. Such a “human sensor” may operate well within a sensor network if he/she would have clearly-defined assignments and constrained services for acquiring and uploading data. In that respect, one may consider client applications with input forms using closed questions and fixed answer lists. By controlling applications on client or server side, the validity of the captured data may be checked as well [6]. We qualify such *iLBS* applications as *formal iLBS. Formal iLBS* can perform well in many application domains such as controlling and sampling procedures [7].

Some studies have mentioned the impact of data gathered via less constrained applications that means that this sensor role comes closer to human way of sensing. Partly analogue examples are known as public authoring [8] and place logging [9]. As such, we make a difference between *formal iLBS* and *informal iLBS*. The latter is based on weakly-defined assignments, having no constrained input procedure and the user-generated content of which is acquired by the in-situ context. *Informal iLBS*-gathered data may be seen as volunteered geographic information (VGI) [10]. The recently-started discussion about quality and credibility of VGI can provide impulse for new research initiatives [5,11,12].

During the organisation of the “Sensing a Changing World” Conference [13], we were challenged to explore data resulting from using an *informal iLBS*. Our starting point was the statement that personal knowledge of outdoor experiences on locations [1,14] may be digitally collected, analysed afterwards and stored in data archives. In this paper, we intended to confirm this statement by exploring these (informal) un-authorized, voluntarily-generated data [5,12]. For our research, we selected data about the cultural history of a location as traceable in the landscape that was recorded by volunteers. We assumed that these volunteers would be devoted to a certain area of interest and have knowledge that may be of interest to others. This knowledge could be triggered associatively on location (*in situ*) and could be registered and stored via *iLBS*. This interactive component added the possibility of finding new anecdotes and facts and discovering hidden layers of information which could then be easily explored. As Butt [15] stated: new *iLBS* will be “about encountering stories on our travels that emerge from and remain tied to specific locations…”.

For the exploration of *informal iLBS*-generated data, we introduced the STEAD approach. This approach involves an experimental methodology by which we attempt to analyse and classify the data acquired. Our intention is to supply these data to cultural-historical data archives. Such archives may serve many *iLBS* applications, *e.g.*, services for recreation, education, and spatial planning that could provide cultural-historic facts, figures and narratives [16–18] about the landscape without having a profusion of signs and information billboards visible in the landscape.

First, we introduce the Digital Dowsing Rod project [19,20] as the STEAD approach arose from this project. The acronym STEAD is the abbreviation of “spatio-temporal *in-situ* experiences as data” and refers to the noun “position”, the verb “stead” and the saying in someone’s stead [21]. Especially the latter seems a link to the “human sensor” that may support personal experiences by information of others.

Secondly, the results of the Digital Dowsing Rod project are used to illustrate the STEAD approach. We used this approach to explore data as sensed via *iLBS*, in order to find out what has been sensed by whom, but also when and where exactly such experiences were recorded and stored [22]. The data analysed by means of these four facets are called Parlance Points (PP). Parlance Points represent points-of-interest (POI) as collected by the “human sensor”. Especially the “who”, “what” and “where” facets will be elaborated upon. Finally, we discuss the results of this experiment and directions for further development.

## 2. ILBS: Digital Dowsing Rod

**Figure 1.**
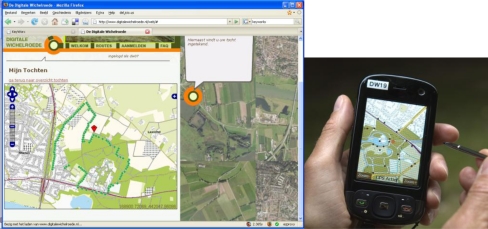
Web browser interface and mobile client interface of iLBS.

In the “Digital Dowsing Rod” [20] project, a consortium developed and tested an interactive location-based service. This service provides cultural-historic landscape information in two ways: from narratives and from facts and figures. The Digital Dowsing Rod (DiWi) is location-based and service-oriented. It can be used to reveal the hidden history of a landscape to visitors and provide them with cultural-historical information that is detectable in the surrounding landscape, using broadly available devices. The HTC P3600 was selected as mobile device. This smart phone (PDA) has GPS, UMTS/HSDPA for data transmission, and audio-video record and display functions. With the pilot application, a “thin” mobile client, users are served by a tailor-made user interface. The user interface resembles information based on different data types. This information consists of maps, walking and biking routes and point-of-interest (POI) locations that are all indicated on these maps, multimedia presentations of cultural-historic information and personal experiences in text, photo, audio and video formats linked to the POIs (Figure 1). By using the smart phone, visitors can explore a landscape and its cultural history using various levels of freedom. This freedom is important because modern tourists are capable of defining their own needs and preferences. They are not always satisfied with the “travel agent’s standard offer”. Modern tourists want to have it all, but not at the same time and not at the same spot. Diversity has become a new keyword in tourism planning [23]. For that reason a DiWi-user can choose from different modes: (a) a predefined trail with historical accounts (b) a personalized route based on personal preferences including related personal historical accounts and (c) a forage through the landscape that takes for granted whatever historical information will be encountered. Furthermore the DiWi application provides the opportunity to record, store and upload personal location based experiences via text processing, voice recording or photo/video camera. Users can generate their own POIs and share them immediately with other users reaching these locations. This makes the DiWi application an *informal iLBS*.

**Figure 2.**
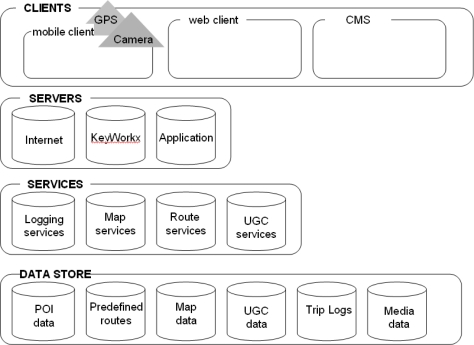
Digital Dowsing Rod Architecture.

Figure 2 shows the outline of the Service Oriented Architecture (SOA) of the Digital Dowsing Rod. This four-tier architecture consists of three clients: (a) web client, (b) mobile client and (c) content management system client (CMS). A web client allows users that have logged in to explore predefined routes, create their own routes, and view routes they have followed before as well as their user-generated POIs. In Figure 1, we see the left side a screen dump of the web client showing a recorded walk. A CMS allows administrators and managers to add and manage predefined POIs, including multi-media files, and to moderate and manage the user-generated content (personal POIs). Through the mobile client, the user can view a map, a route, points-of-interest, and the position of the device. In fact, it promotes interactivity in LBS because it allows users to generate, store and upload their personal text, sound, photo and video files to the data stores.

As Figure 2 shows, all data is stored in various databases (data store tier). For the storage of maps, routes (predefined routes) and networks we used a native data model (Oracle spatial). Data has been implemented as an Open GeoSpatial Consortium [24] compliant Web Mapping Services (WMS) using ArcIMS. All user generated content is stored in the user generated content database (UGC data). The automatically-generated user tracks, from the client-docked GPS whose signals are caught every 15 seconds, are stored in the Trip Logs. All media related either to predefined POIs or user-generated POIs will be stored in the media data. Instead of the term user-generated POIs, from now on we will use the term Parlance Points (PP), as parlance refers to fact, figures and anecdotes that are conventional wisdom.

## 3. Digital Dowsing Rod Case Study

**Figure 3.**
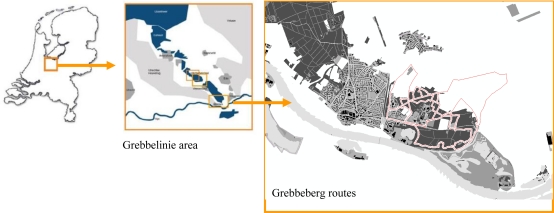
Location of the Grebbeberg site (right) in the Grebbelinie area (middle), The netherlands (left).

The DIWI application was tested in the “Grebbelinie” area (Figure 3) in The Netherlands. This area, originally reserved as a military defense area, exposes fortifications and areas that were inundated in the past. Its use for military purposes lasted from the mid-18^th^ century until World War II. This 60-km long line of defense was demilitarized in 1940, after just five days of war. Many remnants of old battlements are still present today, and some have been reconstructed. Because of the history of heroic actions and battles throughout the centuries, anecdotes are plentiful. Moreover, one also finds beautiful scenery and high biodiversity in the Grebbelinie area.

By means of a media offensive by regional broadcast stations, newspapers, and websites (of newspapers and of cultural-historical organizations), people were informed about the objectives of the project, the landscape history to be studied, and invited to register as test persons. In total, 387 persons applied through the Internet and filled in an on-line questionnaire about their interests and background, to find out to which social and demographic group [25] they belonged, and their level of experience with mobile phone and GPS technology. By the web client they were given the opportunity to select and view routes to prepare a walking or biking trip through the Grebbelinie area. Twenty predefined routes were provided by three sites to start a round trip: Grebbeberg, Renswoude and Scherpenzeel.

Eventually, 168 persons tested the DiWi application in the Grebbelinie during five weekends (Friday to Sunday) in March and April 2008. On whichever weekend day they preferred, the volunteers walked (77%) or biked (20%) a previously-selected route within a time-slot of three hours maximum. At the beginning of their walk or bike trip, they were instructed on how to use the client interface and the options there were to generate their own content. During the instruction session, we stated very clearly that they would be able to record experiences and accounts from their own cultural-historical knowledge of the area.

All trips were GPS-tracked. We even registered all of the people’s interactions with the device when creating Parlance Points during the trip. At the end, the testing group filled in an extensive exit questionnaire about the usability of the application, according to the combined Technological Acceptance Model (*e.g.*, [26,27]) in combination with the Hedonic Information systems approach [28], which was used in the Webpark project [29]. In the end, 150 persons produced valid datasets, which could be used for analysis [20]. The Parlance Points (PPs) that were generated by the user, such as recorded texts, pictures and videos, were geo-coded by latitude and longitude. These PPs represented the personally-experienced cultural history of the landscape. The PPs from the DiWi application test were used in this paper to illustrate the STEAD approach and discuss the “human sensor”.

## 4. The STEAD Approach

We developed the STEAD approach for the exploration of *iLBS* user-generated content and is inspired by previously published ideas [15,18,22]. The data exploration by the STEAD approach may support LBS applications dedicated to recreation, education and spatial planning. In this respect an important outcome is the discovery of generated user content that is different and distinctive [11] and may be used to construct narratives for outdoor experiences.

Central to STEAD is that the nature of the data be recognized using the facets (a) who, (b) what, (c) where, (d) when and their interaction [2,3,22]. The latter is important if narratives are to be constructed from the data. In this section, we present the first three facets (who, what, and where), each one illustrated by data from the DiWi project. The fourth facet and the interactions are discussed in the conclusions.

### 4.1. Who sensed

The demographic and social class to which the human sensor belongs can give a first impression of the validity of the user-generated data for re-use. A description of the relationship between user-generated content and the user’s age, gender, lifestyle [25,30] landscape experience as presented in studies of Kaplan and Kaplan, such as [31], form a first step in the analysis PPs.

In the DiWi test, 67% of the registered DiWi applicants were male and 33% female. Most of them had higher education and the average age was 48. This age number is of special interest for the “what” facet (see below). The applicants were divided into eight mentality classes [25] (Table 1). Mentality classes represent the diversity of Dutch society according to lifestyle and personal attitude. The mentality classes are originally based on a panel of approximately 95,000 Internet respondents. The table indicates that DiWi appeals mostly to three mentality groups: cosmopolitans, post materialists and postmodern hedonists. These mentality groups differ significantly from the average mentality class distribution in The netherlands (Table 1).

The motives of the DiWi test persons for recreation appeared to be different than that of the general Dutch population (Table 2). Their interest in the landscape’s cultural-history was particularly striking.

Of the 168 testers, 63% chose the Grebbeberg site (Figure 3) to use the *iLBS* and 70% of this group selected a predefined walking route of three or five kilometers. For the other two sites Renswoude and Scherpenzeel, 67 and 64% of the participants walked a predefined route, respectively.

The DiWi results, such as demographics, mentality class and interest, indicated that the test group represented a context-dedicated group.

### 4.2. What was sensed

Within the given context, the “what” facet was a leading item. The meaning of the data generated via an *informal iLBS* can be difficult to define because of the variety of data formats, ranging from text, pictures and photos to sound and video files. In the DiWi project we dealt with that same problem, because the PPs had been captured in many formats. Using the STEAD approach, we started by classifying all PPs into text, picture and video classes (level 1). Every type of format specifies a form of expression (written, pictorial, musical and/or movie language). In that respect, languages could express the *Off* and the *About* meaning [22].

In this paper we focus on the *Off* meaning classification of pictures. Oku *et al* [32] stated that pictures can be divided into three classes: place, event and object. A place picture presents a specific area not containing a specific item, an event picture shows people and the activities in which they were engaged, and an object picture presents specific items such as flowers, a distinctive tree, a waterfall or an artificial structure. Oku showed that older (>40 years) people concentrate mainly on “place” type pictures and took relatively few “event” pictures. In the STEAD approach these place and object pictures are of interest.

The DiWi case study showed [19] that the testers had cultural-historical interest. We did not limit the selection of the originally stored 345 PP beforehand, and accepted that all PPs show evident historic features. In general, every person stored on average three PPs during his or her trip. Of the total number of PPs, 28% were text, 6% video and 67% photographs.

Inspired by Daniel [33], Hunter [34] and Snavely [35] we assumed that a picture represents the perception of a person intending to frame [16] a certain cultural-historical feature in a location within a landscape, because of the recognition of a specific historic episode. This assumption leads into a second classification. We labeled the pictures into place, event and object pictures. To illustrate this classification by the DiWi results, we classified 231 pictures (for examples, see Figure 5). According to Oku’s classification and knowing the average age (48 years) of the DiWi test persons, we can say that most pictures (79%) dealt with places and objects.

After a closer look, based on classification into place, event and object-oriented pictures. Events were shown in 21% of the pictures, and 35% were photographed objects such as animals and plants (Table 3). The other photographs (44%) showed place oriented pictures.

We classified 38% as relevant to cultural-historical interest. This meant 0.8 PP pictures per person. The pictures in the class of relevance for cultural history belonged mainly to events and objects (the latter is mainly dominated by pictures of animals and plants).

**Figure 4.**
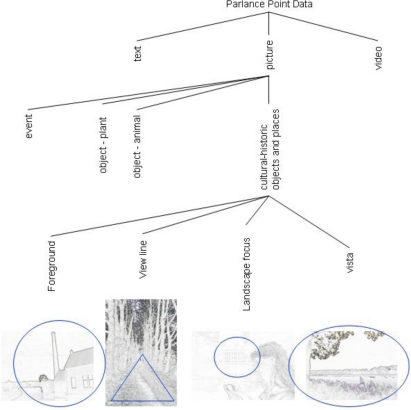
Three levels of PP classification. The pictures on the third level show the four framing principles.

**Figure 5.**
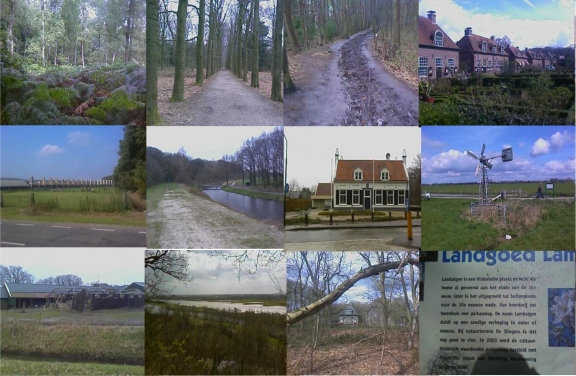
Examples of PP pictures made by test persons. Left to right in the 1^st^ row: vista, view line, foreground, focus; 2^nd^ row: focus, view line, foreground, focus; 3^rd^ row: foreground, vista, focus, other.

Afterwards, the framing of the cultural-historic feature in the picture was classified as foreground (objects according Oku *et al.* [32]), landscape foci, view lines and vistas (Figure 4). This classification may be compared with Litton’s [36] feature landscape, focal landscapes, enclosed landscapes and panoramic landscapes.

On classification level 3 (Table 3) we found 121 photographs of interest: 39% of these photographs shows foreground objects, mainly details of buildings and 17% represent landscape foci, especially buildings (*e.g.*, old farmhouses, castles) in the landscape. View lines such as an avenue or a watercourse were found on 18% of the photographs. The last 26% were vistas, wide views over an open area or from a landscape panorama viewpoint.

This facet of the STEAD approach enabled us to classify the framing of the PP pictures, but we must emphasize that we only addressed the *Off* meaning, and not the *About*, of pictures as sub-classes of PPs.

### 4.3. Where has been sensed

Besides the who and what facet, the “where” facet is of great importance for updating or developing *iLBS* applications that may benefit from PPs. Analysing the spatial distribution of locations of PPs gives insights into what locations are described, how often they have been described and how close to other PPs and predefined routes they are located. This may help to identify more or less favourite locations that could help to discover new and distinctive PPs as well as other historical accounts.

To illustrate the analysis of this facet, we selected 158 PPs related to five predefined routes of three and five kilometers in the Grebbeberg site. These PPs were sensed during 70 walking trips along these routes. It means that every person sensed on average 2.25 PPs per tracked trip.

The spatial density of PPs was determined by transformation of PP data into raster cells having different spatial resolutions. The resolutions used were 4, 8, 16, 24, 32, 40, 48, 56, 64, etc, up to 256 meters. Each raster cell showed the number of PPs per areal unit. We chose for this method because it is known that the spatial accuracy of built-in GPS receivers under experimental conditions is within 2 meters [37].

**Figure 6.**
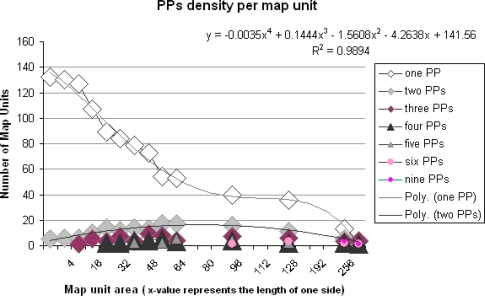
Parlance Points per map unit.

Figure 6 shows 132 units with just one single PP (one unit) by a tiling of the study area in mapping units of two square meters. A trend analysis (4^th^ order polynomial, see Figure 6) shows a decline of single PP map units in relation to the increase of the map unit size. A division of the area into 256 by 256 meter map units shows less than 20 single PP map units. The trend line (2^nd^ order polynomial) of two PPs per map unit showed a small increase just near to 20 units and then a decrease. However we should take into account that only 0.2% of all map units (8 by 8 meters) represent PP locations. This share increased to 20% for 128 by 128 meters map unit size

The PP density per map unit per site gives an impression of the uniqueness of PPs (Figure 6) as shown by the one PP line for map units greater than 96 square meters. It also provides a way to discuss the granularity of an *iLBS* via the stack of PP densities per map unit, such as the stack PP densities for 64 meters granularity (Figure 6). The stack is shown by two types of triangles representing four and five PPs, and three types of rotated squares representing one, two and three PPs.

**Figure 7.**
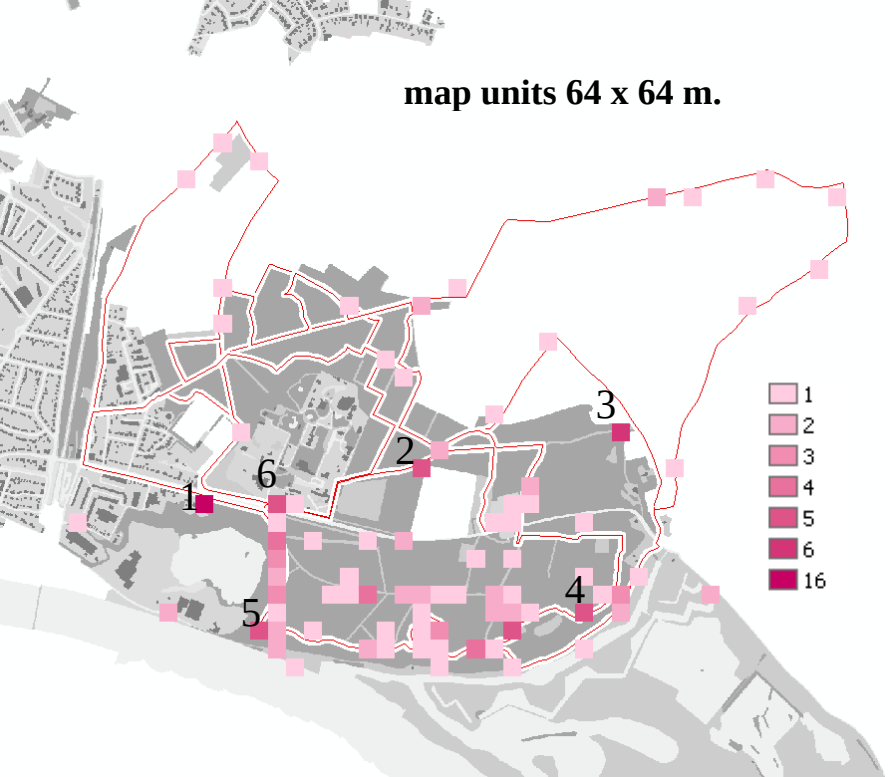
PP density per map unit (64 × 64 meters).

Regarding the density of PPs, we found that within map units ranging from 2 by 2 meters up to 64 by 64 meters, on average 93 units consist of 1 PP, 11 units consist of two PPs, four units consist of three PPs, two units consist of four PPs and one unit consists of five PPs (Figure 7). According to the spatial distribution of the map units with one and more PPs, we noticed that some map units have a higher density than others. The density number could give an idea of the more and less informative places as well as their uniqueness. Especially the locations 1, 2, 3, 4, 5 and 6 in the figure evoked more clues (four up to 16 PPs per cell) for recording experiences.

If we relate the units with high densities to the “what” facet of the PP, then we find in unit 2 five PPs that represent view lines (avenues). The five PPs linked to the map units 3, 4 and 5 show vistas (landscape panoramas) for which this edge of the area is famous. The map unit at location 6, the start location, stored another five PPs showing mainly tests made by the users as part of the instruction session at the beginning of a walking trip. Location 1 with 16 PPs is an outlier because of GPS error.

For analysis of the relationship between PPs and routes, the five predefined routes have been merged and subsequently buffered by 2, 4, 8, 16, 24, 32, 40, 48, 56 and 64 meters diameter around the centre line of the route. Subsequently, a spatial overlay between PP point data and the buffer zones was used to give an insight into the relationship between distances of PP locations and the location of predefined routes.

**Figure 8.**
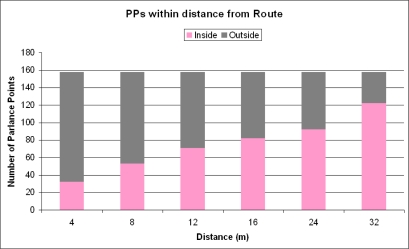
Distance relation between PP location and predefined routes.

The results of the distance from the routes analysis show a linear relation between the number of PPs within a certain distance of the center line of the predefined routes. Fifty percent of the PPs were located inside the buffer zone of 8 meters on either side of the route (see X-value 16, Figure 8). An area of 16 meters on both sides of the route contained nearly all PPs (see X-value 32 in Figure 8). These numbers show that PPs acquired by the DiWi volunteers were close to the predefined routes.

## 5. Discussion and Conclusions

**Figure 9.**
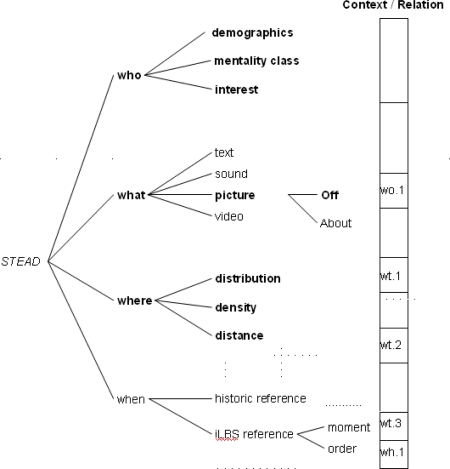
Overview of the STEAD approach (**bold**: presented by the paper).

We set up the STEAD approach because we were challenged by the idea of the human sensor. This approach intends to provide information about what has been sensed by whom, where and when. This may support the update, extension and further development of points of interest for *iLBS*. An overview of the intended approach, with the bold characters showing the who, what and where facets that were addressed and illustrated in this paper is given in Figure 9. The figure makes clear that some facets have not been addressed at all (“when” facet) or partly addressed.

In this paper, we only examined the relation between “who” and “what” (*wo.1* in Figure 9) using the findings of Oku [32], as well as the relationship between density and distribution of pictures and their framing (*wt.1* in Figure 9). However, many other relationships would need to be analysed before PPs show their credibility as points of interest, and become part of a narrative. In that case, the *About* meaning of PPs plays a major role, but has not been addressed in this paper. For example, the previously-presented relationship between PP density and framing shows that many PPs could have the same meaning; therefore the fact that a certain location may have one specific landscape historical highlight makes evident why it is unique.

Regarding the human sensors themselves, the “who” facet, it is important to find out to which social and demographical groups they belong. In our DiWi example, we dealt with a group of people that belong to three mentality groups. They were well-educated and their average age was above 45 years. The participants carried out the work diligently and along the lines of cultural-historical interest in the landscape. It has been shown that for the whole study area, 2.1 PPs per person and for the Grebbeberg site alone, 2.25 PPs per person were recorded. However we could not verify the involvement statistically due to the lack of personalized PP data. For the “what” facet, we defined a three-level classification system to describe the *Off* issue. The DiWi case showed that approximately 38% of the PPs were dedicated to the cultural history of the landscape. This means that the human sensor in this study, however dedicated, recorded many other topics as well. Moreover, the framing of sensed cultural historical items showed a preference for foreground and vista frames.

Of all test persons, 54% added PPs. The testers who did not add content had various reasons for not doing so [19]: 29% just did not feel the need to add content, 17% experienced it as too complicated and 7% blamed the poor weather conditions. There were neither statistical relationships found between the amount or type of PPs and recreational motives of the mentality groups, nor with age or gender. Of all the testers, 74% appreciated the idea of adding information themselves.

The “where” facet was addressed by distribution, density and distance of PPs. Given a predefined route in the DiWi experimental setting, we found that 77% of the PPs were located within a distance of 16 meters from the centerline of a route and could be explained by a linear function. In that respect, the “human sensor” seems rather fixed. Regarding density, it is obvious that some locations provided more attractive features than other. The size of the map unit strongly influenced the spatial distribution of the map units with high or low PP densities. In our case the PP densities ranged from 1 to 5 per map unit size calculated for map unit sizes of maximally of 64 by 64 meters. A map unit size of 256 by 256 meters showed a maximum of 12 PPs per map unit. We found that the number of locations with a unique PP per map unit significantly diminished according to a polynomial trend line. We have not validated our statement by a random realization of PP densities.

The “when” facet has not been addressed in this approach. However, studying this facet could result in useful information because the GPS information time stamps every recorded PP. We label this as the *iLBS reference* (Figure 9). This time stamp provides insight into the moment, “densities” of recording moments, and the order of recordings moments. The former tells more about the temporal variation of location visits and the latter may be helpful to reconstruct the context, because a previous recording will probably influence the follow-up, *in situ* recording. Besides the *iLBS reference,* we would also need to classify the specific historical period or episode that is represented by the PP. In the STEAD approach, we label this the *historic reference*.

At this time, it is obvious that we must study more literature from various disciplines to expand the STEAD approach, in order to find methods that could support the analysis of the different facets and their relationships, continue evaluating our first quantitative findings and use classified PPs in many types of location-based services, preferable the ones in favour of narratives. For now, we conclude that interest in this type of human-sensed data (PPs) requires *iLBS* experiments to be carried out by dedicated users, along pre-defined routes and based on expert-classified landscape units. These topics - including relationships between the facets - could then become part of the exploration of *iLBS* user-generated content. Also the (semi)automated classification of PPs can be studied. In this study, for example, the classification of pictures was time-consuming. Ongoing research [10,38–40] based on the availability of digital photographs via Internet, will bring forward a new wave of classification algorithms and applications that should support such (semi)automation.

Some final remarks can be made on the role of user-generated data as a source for intended data archives. In the DiWi-project, the volunteers were asked how many PPs they downloaded during their trip and how they assessed the quality of these PPs. Of the testers, 62% downloaded user-generated content to see what others had “left behind”. Because 47% of the user-generated content appeared to be of a personal nature (events and objects), the assessment was not very positive. The testers did not agree with the proposition that user-generated content is interesting and as such, an added value to explore and learn more about the region. In contrast, the assessment showed that for personal use (*e.g.*, reviewing their own trip on the website) the user-generated content (especially events and objects) have an additional value. From those results, it appeared that user-generated content can best be divided into the classes mentioned by Oku [32]. However, we stress that a context must be given to information that is acquired. The ideas of Cloke [16], Potteiger [17] and Spirn [18], about landscape narratives could play an important role in ongoing studies. It is for that reason that relations between the four facets may become crucial in respect to the use of the sensed data in *iLBS* applications. Critical analyses of PPs categories may reveal that certain historical features or sites have become duplicated so often that they risk becoming a cliché. This may have consequences for general historical comprehension and the credibility of PPs, as landscapes may become full of human sensors.

## Figures and Tables

**Table 1.** Distribution of mentality classes: comparison between all registered Diwi applicants (Diw-registered) and the Diwi-testers, and Dutch citizens.

**Mentality-group**	**Diwi-registered (in %)**	**Diwi-testers (in %)**	**Dutch (in %)**
Traditional middle class	9	3	18
Convenience oriented	3	1	9
Modern middle class	6	7	22
New conservatives	5	5	8
**Cosmopolitans**	**18**	**25**	10
Social climbers	4	3	13
**Post materialists**	**32**	**35**	10
**Post modern hedonists**	**19**	**20**	10
No data	3	1	

**Table 2.** Recreational motives of theDiwi testers (%) compared to average Dutch citizens.

**Motif**		
	**Diwi-testers (in %)**	Dutch* (in %)
Social interaction	19	27
Diversion	22	34
Interest	**27**	14
Experiencing a different world	14	15
Physical challenge	18	9

• source: [19]

**Table 3.** Parlance points classification.

**Level 1**	**Parlance Points**			

N = 345	photographs	text	video	audio
	231/67%	95/28%	19/6%	*Not supported*

**Level 2**	**Picture Styles**			

N = 231	place	event	**object** (including plants/animals)	
	102/44%	48/21%	81/35%	
	relevant	non relevant		
	132/57%	99/43%		

**Level 3**	**Framing classes**			

N = 121	Foreground (object)	Landscape focus (place)	View line (place)	Vista (place)
	51/39%	22/17%	24/18%	35/26%
